# A multi-center, open-label, observational study to evaluate the efficacy and safety of LivaloZet^®^ tablets in patients with dyslipidemia and metabolic syndrome: PIVOT study protocol

**DOI:** 10.3389/fendo.2026.1862394

**Published:** 2026-07-10

**Authors:** Kyong Hye Joung, Bon Jeong Ku

**Affiliations:** 1 Department of Internal Medicine, Chungnam National University College of Medicine, Daejeon, Republic of Korea; 2 Department of Endocrinology & Metabolism, Chungnam National University Sejong Hospital, Sejong, Republic of Korea; 3 Department of Endocrinology & Metabolism, Chungnam National University Hospital, Daejeon, Republic of Korea

**Keywords:** dyslipidemia, ezetimibe, metabolic syndrome, observational study, pitavastatin, real-world evidence

## Abstract

**Introduction:**

Dyslipidemia accompanied by metabolic syndrome substantially elevates cardiovascular risk by clustering multiple risk factors, including abdominal obesity, hypertension, hyperglycemia, and atherogenic dyslipidemia. The prevalence of both conditions is rising in Korea, underscoring the need for effective and metabolically safe lipid-lowering therapy. LivaloZet^®^ Tablet (pitavastatin/ezetimibe fixed-dose combination) addresses this need by combining complementary cholesterol-lowering mechanisms with the glucose-neutral profile of pitavastatin.

**Methods and analysis:**

The PIVOT study is a multi-center, non-interventional, prospective observational study enrolling up to 10,000 Korean adult patients with dyslipidemia accompanied by metabolic syndrome who have been prescribed LivaloZet^®^ Tablets (pitavastatin/ezetimibe 2/10 mg or 4/10 mg) by their treating physician as part of routine clinical care. Metabolic syndrome is defined according to the modified NCEP-ATP III criteria for the Asian population. Assessments are conducted at baseline, Week 24, and Week 48. The primary endpoint is the percent change in low-density lipoprotein cholesterol (LDL-C) from baseline at Week 24. Secondary endpoints include LDL-C changes at Week 48, changes in the full lipid panel, and LDL-C target achievement rates by cardiovascular risk group. Exploratory endpoints encompass glucose metabolism parameters (HbA1c, HOMA-IR, HOMA-β), medication adherence and persistence, and high-sensitivity C-reactive protein. Safety is assessed through adverse events, laboratory tests, and vital signs, including systematic monitoring of AST, ALT, and creatine kinase for statin-related hepatotoxicity and myopathy. The study was approved by the Institutional Review Board of Chungnam National University Hospital (2025-07-065) and is registered at ClinicalTrials.gov (NCT07523971).

**Discussion:**

The PIVOT study will generate prospective large-scale real-world evidence on the LDL-C-lowering effectiveness and safety patterns associated with use of a pitavastatin/ezetimibe fixed-dose combination in Korean patients with dyslipidemia accompanied by metabolic syndrome. The inclusion of glucose metabolism and inflammatory markers will provide descriptive information on the metabolic safety profile of this regimen. As a non-randomized, single-arm observational study, this design cannot establish causal effects or comparative efficacy versus other lipid-lowering regimens. Findings should be interpreted descriptively, acknowledging potential confounding by indication, selection bias, and inter-laboratory variability.

## 1 Introduction

Dyslipidemia is defined as a condition in which low-density lipoprotein cholesterol (LDL-C) is elevated, triglycerides (TG) are elevated, or high-density lipoprotein cholesterol (HDL-C) is reduced in the blood (1). Since dyslipidemia acts as a major risk factor that can lead to cardiovascular disease and progress to life-threatening conditions, managing blood lipid levels is of critical importance (1–4). In particular, patients with dyslipidemia accompanied by metabolic syndrome carry multiple cardiovascular risk factors - including obesity, hypertension, hyperglycemia, and various types of dyslipidemia - making rigorous lipid management essential for the prevention of cardiovascular disease and improvement of prognosis (1, 5, 6).

According to the recently published 2024 Dyslipidemia Fact Sheet and Metabolic Syndrome Fact Sheet, the prevalence of dyslipidemia among Korean adults aged 20 years and older was 37.5%, while the prevalence of metabolic syndrome among Korean adults aged 19 years and older was 24.9%; both conditions showed an increasing trend in prevalence with age (7, 8). Considering the rapid aging of Korea’s population, the number of patients with dyslipidemia accompanied by metabolic syndrome is expected to continue rising, making aggressive lipid control increasingly important (9).

Among the various lipid-lowering agents used in pharmacotherapy for dyslipidemia, statins are generally administered once daily, offer excellent LDL-C-lowering efficacy, and have a relatively favorable safety profile, making them the first-line treatment (9). Statins competitively inhibit the reductase of 3-hydroxy-3-methylglutaryl-coenzyme A (HMG-CoA), a cholesterol precursor, thereby reducing hepatic cholesterol synthesis, upregulating LDL receptor expression, accelerating hepatic uptake of circulating cholesterol, and decreasing plasma total cholesterol (TC) and very low-density lipoprotein (VLDL) (9, 10). However, concerns exist about dose escalation of statins until LDL-C reaches target levels, due to safety issues including dose-dependent hepatotoxicity and the risk of rhabdomyolysis (11). Therefore, in patients requiring a potent LDL-C reduction or in whom high-dose statin therapy is not feasible due to adverse effects, combination therapy with ezetimibe is recommended (9, 12, 13).

Ezetimibe is a selective cholesterol absorption inhibitor that acts on the Niemann-Pick C1-Like 1 (NPC1L1) protein, a cholesterol transport carrier in the intestinal villi, without affecting the absorption of bile acids, fatty acids, triglycerides, or fat-soluble vitamins, thereby inhibiting the absorption of both dietary cholesterol and biliary cholesterol undergoing enterohepatic circulation (14, 15). As monotherapy, ezetimibe reduces LDL-C by approximately 17–20%, which is weaker than statins; however, when combined with a statin, complementary effects arise from the two distinct mechanisms of action - namely, statin-mediated inhibition of endogenous cholesterol synthesis and ezetimibe-mediated inhibition of exogenous cholesterol absorption (15–20). Given these advantages, combination therapy with a statin and ezetimibe has been deemed effective and safe for reducing LDL-C levels, leading to the development and commercialization of multiple fixed-dose combination products.

The product evaluated in this observational study, LivaloZet^®^ Tablet, is a fixed-dose combination of pitavastatin and ezetimibe that was approved and marketed in Korea in 2021 for primary hypercholesterolemia. LivaloZet^®^ Tablet is a lipid-lowering agent indicated for patients requiring potent LDL-C reduction or those for whom high-dose statin therapy is not feasible due to adverse effects. Furthermore, since pitavastatin is known to have a relatively neutral adverse effect profile on glucose metabolism compared with several other statins (21–23), LivaloZet^®^ Tablet is expected to be a useful therapeutic option for lipid management in patients with metabolic syndrome who have or are at risk of hyperglycemia (21–23). Therefore, this study aims to describe the LDL-C-lowering effectiveness and safety profile of LivaloZet^®^ Tablet in Korean adult patients with dyslipidemia accompanied by metabolic syndrome under real-world conditions.

## 2 Methods and analysis

### 2.1 Study design

This study is a multi-center, non-interventional, prospective observational study conducted in routine clinical practice. As a non-interventional study, treatment decisions—including drug selection, dose, dose changes, and discontinuation—are made by the treating physician independently of the study, in accordance with their usual clinical judgment and the approved product label. The study participants are patients with dyslipidemia accompanied by metabolic syndrome. The objective of this study is to describe the LDL-C-lowering effectiveness and safety profile associated with the use of LivaloZet^®^ Tablets in patients with dyslipidemia accompanied by metabolic syndrome in real-world Korean clinical settings. The products under evaluation are LivaloZet^®^ Tablets 2/10 mg (pitavastatin calcium/ezetimibe 2/10 mg) and LivaloZet^®^ Tablets 4/10 mg (pitavastatin calcium/ezetimibe 4/10 mg). Dose selection (2/10 mg vs 4/10 mg) is made by the treating physician based on the patient’s cardiovascular risk category, baseline LDL-C level, prior lipid-lowering treatment history, and clinical judgment. Dose modifications, including up-titration, down-titration, or discontinuation, are permitted at any time according to routine clinical practice and will be recorded.

**Figure 1:**
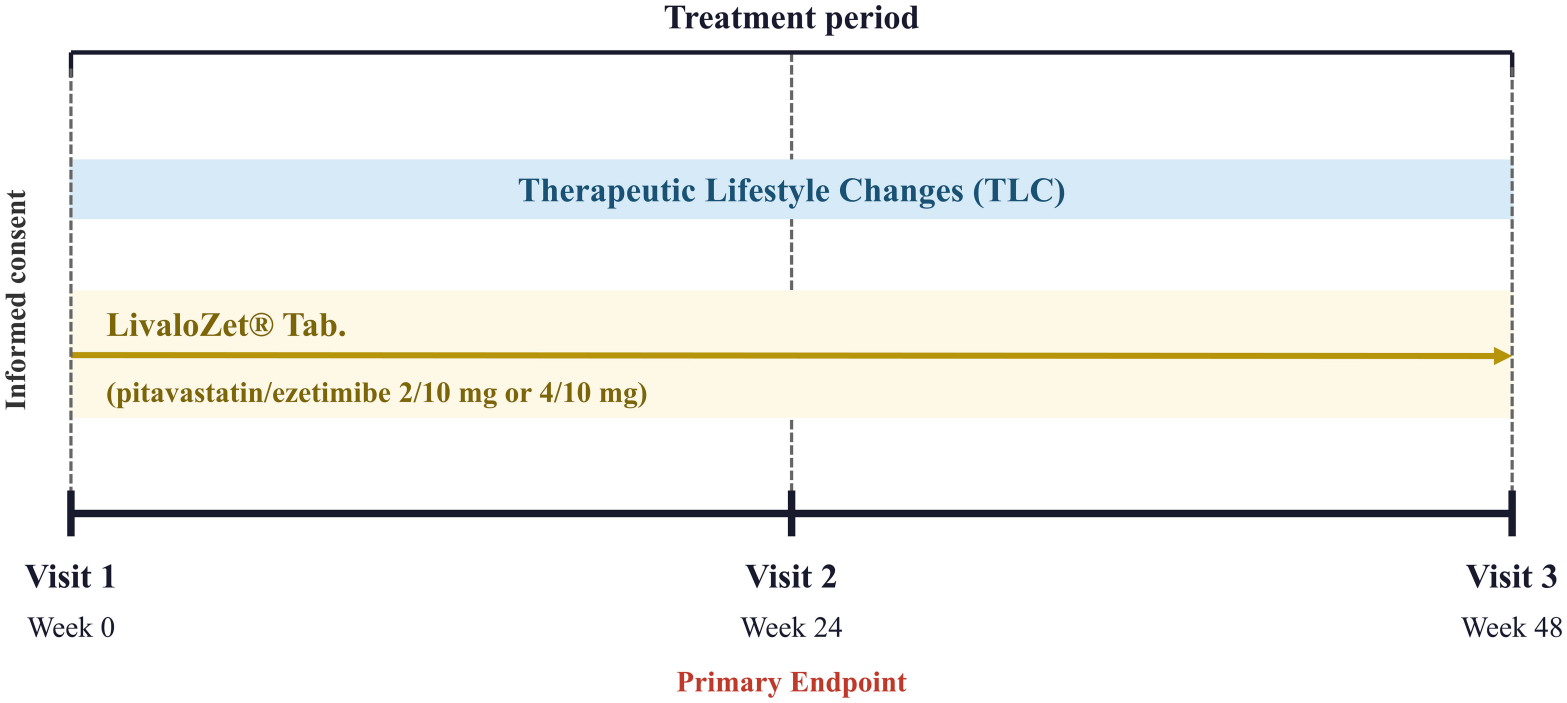
The flowchart of the study.

The flowchart of the study is shown in **Figure 1**. Participants who provide written informed consent will be assigned a participant identifier and assessed for eligibility based on the inclusion and exclusion criteria. Those eligible based on the inclusion and exclusion criteria will have scheduled visits at Week 24 (Visit 2) and Week 48 (Visit 3) from the baseline visit (Visit 1). Participants with any unresolved adverse events (AEs) at the end of study (EOS) visit will be followed until resolution, stabilization, or for a minimum of 4 weeks after the EOS visit, whichever is later, with extended follow-up at the investigator’s discretion for serious or clinically significant events. A detailed schedule of enrollment and assessments is presented in **Table 1**.

**Table 1:** Participant timeline: schedule of enrollment and assessments.

Visit	Visit 1 (Baseline)	Visit 2	Visit 3 (EOS)	UV
Day (Week)	**D1 (W0)**	**D169 (W24)**	**D337 (W48)**	**-**
Visit window	**-**	**-2M~+1M**	**-2M~+1M**	**-**
Written informed consent¹)	●			
Demographic information²)	●			
Baseline disease information³)	●			
Cardiovascular risk classification	●			
Medical history^4^)	●			
Prior/concomitant medication^5^)	●	●	●	○
Physical examination	●	●	●	○
Vital signs (blood pressure, pulse rate, body temperature)	●	●	●	○
Physical measurement^6^)	●	○	●	○
Serum lipids^7^)	●	●	●	○
Blood glucose and glucose metabolism parameters^8^)	○	○	○	○
Cardiovascular risk biomarker (hs-CRP)^9^)	○	○	○	○
Blood chemistry (AST, ALT, CK)¹^0^)	●	●	●	○
Apolipoprotein B (ApoB)¹¹)	○	○	○	○
TLC education and checking	●	●	●	○
Inclusion and exclusion criteria checking	●			
Participant enrollment	●			
IP prescription/administration checking	●	●	●	○
Medication adherence & persistence¹²)		●	●	○
AEs¹³)		●	●	○

AEs, Adverse events; EOS, end of study; TLC, Therapeutic Lifestyle Change; UV, unscheduled visit.

**●** To be conducted in all participants enrolled in the study.

**○** Optional items for which information will be collected only if test results are obtained in routine clinical practice. Information will not be collected if there are no test results.

^1)^All study related procedures should be performed after written informed consent is obtained; After informed consent is obtained, a participant identifier will be assigned.

^2)^Date of birth, age, sex, pregnancy/breastfeeding, and smoking history will be collected.

^3)^Date of initial diagnosis of dyslipidemia/metabolic syndrome, presence of pre-diabetes/diabetes, date of initial diagnosis of diabetes, family history of premature coronary artery disease, presence of hypertension, and presence of target organ damage will be collected. If classification of cardiovascular disease risk groups has been previously performed, or if the necessary information for classifying cardiovascular disease risk groups is available, allowing the investigator to classify the risk groups directly, the relevant information will be collected. Cardiovascular risk classification (per **Table 2**) will be assigned at Visit 1 by the treating investigator (Section 2.3) and will not be re-assigned during follow-up.

^4)^Medical history (including surgical/procedure history) within 12 weeks prior to Visit 1 will be collected. For medical history relevant to the inclusion and exclusion criteria, information throughout lifetime will be collected, unless otherwise specified.

^5^Medication information on lipid regulators, antidiabetics, and other drugs will be collected. Drugs administered within 4 weeks prior to Visit 1 will be collected as prior medication. If there is a history of treatment with lipid regulators within the predefined duration of information collection for prior medication, medication information on lipid regulators within 8 weeks prior to Visit 1 will be collected. In addition, if results of prior serum lipid or blood glucose and glucose metabolism parameter tests are used as baseline (Visit 1) results, information on lipid regulators will be collected in the same manner as described above based on the time point of the tests. Drugs administered from after the first study drug dosing to the end of observation will be collected as concomitant medication.

^6)^Height, weight and waist circumference will be measured, and body mass index (BMI) will be calculated. Height will be measured at Visit 1 only.

^7)^LDL-C, TC, TG, HDL-C, and non-HDL-C will be tested. LDL-C may be measured either directly (by homogeneous direct assay) or calculated using the Friedewald formula; when TG ≥400 mg/dL, direct LDL-C measurement is required (Section 2.7). If test results obtained within 4 weeks prior to Visit 1 are available and the participant had no treatment with lipid regulators within 4 weeks prior to the test or was treated with lipid regulators using the same dosage and administration for at least 8 weeks, the test results may be used as baseline (Visit 1) results. Before testing, a blood sample is to be collected after at least 12 hours of fasting, if possible; If it is difficult for the participant to remain fasted for 12 hours, at least 9 hours of fasting should be maintained.

^8)^If test results for HbA1c, HOMA-IR, and HOMA-β are available, the information will be collected. If it is not feasible to collect HbA1c test results within the visit window in patients with pre-diabetes or diabetes, HbA1c testing will be performed. If test results obtained within 4 weeks prior to Visit 1 are available and the participant had no treatment with lipid regulators within 4 weeks prior to the test or was treated with lipid regulators using the same dosage and administration for at least 8 weeks, the test results may be used as baseline (Visit 1) results. Before testing, at least 8 hours of fasting should be maintained.

^9)^If test results for hs-CRP are available, the information will be collected. If test results obtained within 4 weeks prior to Visit 1 are available and the participant had no treatment with lipid regulators within 4 weeks prior to the test or was treated with lipid regulators using the same dosage and administration for at least 8 weeks, the test results may be used as baseline (Visit 1) results.

^10)^Aspartate aminotransferase (AST), alanine aminotransferase (ALT), and creatine kinase (CK) are collected systematically at every visit to monitor statin-related hepatotoxicity and myopathy. If test results obtained within 4 weeks prior to Visit 1 are available, they may be used as baseline (Visit 1) results. Pre-specified safety thresholds for action: AST or ALT >3× upper limit of normal (ULN); CK >5× ULN with muscle symptoms, or >10× ULN regardless of symptoms (Section 2.4.3).

^11)^Apolipoprotein B (ApoB) is not a required measurement; it will be collected and analyzed descriptively only when available from routine clinical practice (Section 2.4.2).

^12)^Medication adherence (proportion of days covered, PDC), persistence (time from initiation to discontinuation), dose modifications, and treatment switching will be collected at follow-up visits (Section 2.4.2).

^13)^All clinically significant medical conditions or abnormalities occurring from the first study drug dosing to the EOS visit will be collected as AEs. Any unresolved AEs at the time of the participant’s EOS visit will be followed until resolution, stabilization, or for a minimum of 4 weeks after the EOS visit, whichever is later, with extended follow-up at the investigator’s discretion for serious or clinically significant events (Section 2.1).

The study protocol was approved by the ethical review board of Chungnam National University Hospital with the certificate number of 2025-07-065. This study was registered at www.clinicaltrials.gov (NCT07523971). The study protocol was developed in accordance with the SPIRIT 2013 guideline (for protocol items relevant to observational designs) and is reported according to the STROBE statement for observational studies.

### 2.2 Participants

#### 2.2.1 Definition of dyslipidemia and metabolic syndrome

Dyslipidemia is defined according to the 2022 Korean Society of Lipid and Atherosclerosis (KSoLA) guidelines as the presence of any one or more of the following: LDL-C ≥160 mg/dL, TC ≥240 mg/dL, HDL-C <40 mg/dL, or TG ≥200 mg/dL (9). Metabolic syndrome is defined according to the modified NCEP-ATP III criteria for the Asian population as the presence of three or more of the following five components: (1) abdominal obesity (waist circumference ≥90 cm for men or ≥85 cm for women, based on Korean cutoffs); (2) elevated triglycerides (≥150 mg/dL or on lipid-lowering therapy for hypertriglyceridemia); (3) reduced HDL-C (<40 mg/dL for men, <50 mg/dL for women); (4) elevated blood pressure (systolic ≥130 mmHg or diastolic ≥85 mmHg, or on antihypertensive therapy); and (5) elevated fasting glucose (≥100 mg/dL or on antidiabetic therapy).

#### 2.2.2 Inclusion criteria

To be eligible for this study, participants must meet all of the following criteria.

1. Age ≥19 years old at the time of written informed consent.
2. Participants diagnosed with dyslipidemia and metabolic syndrome as defined in Section 2.2.1.
3. Participants who had no treatment history with lipid regulators within 4 weeks prior to Visit 1 or who had insufficient therapeutic effects from ≥8 weeks of treatment with lipid regulators using the same dosage and administration (these two subgroups—treatment-naïve and inadequate-response—will be pre-specified and analyzed separately as a key subgroup analysis, as they may exhibit differential LDL-C response).
4. Participants who meet the following LDL-C levels according to the classification of cardiovascular disease risk groups (9) (**Table 2**) at Visit 1 or who are planned to be treated with LivaloZet^®^ Tablets based on the judgment of the investigator.
5. Participants who voluntarily sign the informed consent form for study participation.

**Table 2:** Classification of cardiovascular risk groups and target LDL-C level.

Risk group	Criteria for classification	LDL-C (mg/dL)
Very high risk group	Coronary artery disease	≥ 55
High risk group	Atherosclerotic ischemic stroke, transient ischemic attack, carotid artery disease, peripheral arterial disease, abdominal aortic aneurysm, diabetes mellitus (duration of disease ≥10 years; or concurrent major cardiovascular disease risk factors† or target organ damage‡)	≥ 70
Diabetes group	Diabetes mellitus (duration of disease <10 years, no major cardiovascular disease risk factors†)	≥ 100
Moderate risk group	≥2 major cardiovascular disease risk factors†	≥ 130
Low risk group	≤1 major cardiovascular disease risk factor†	≥ 160

† Major cardiovascular disease risk factors (1): Age: ≥ 45 years old in men, ≥ 55 years old in women; (2) Family history of early onset coronary artery disease: Parents or siblings who had an onset of coronary artery disease at the age of <55 years in men and <65 years in women; (3) Hypertension: Systolic blood pressure (SBP) ≥ 140 mmHg or diastolic blood pressure (DBP) ≥ 90 mmHg or treatment with antihypertensives; (4) Current smoking; (5) High density lipoprotein-cholesterol (HDL-C) < 40 mg/dL (If HDL-C ≥ 60 mg/dL, 1 will be subtracted from the number of major risk factors).

‡ Target organ damage: Albuminuria (including microalbuminuria), chronic kidney disease (estimated glomerular filtration rate [eGFR] < 60 mL/min/1.73 m²), retinopathy, neuropathy, left ventricular hypertrophy.

LDL-C, low-density lipoprotein cholesterol.

#### 2.2.3 Exclusion criteria

Individuals who meet any of the following criteria cannot participate in the study.

1. Individuals for whom LivaloZet^®^ Tablets is contraindicated as specified in the label’s Precautions for Use.
2. Individuals who were treated with other investigational products (IP) or investigational devices within 4 weeks prior to participation in the present study or who are expected to have such treatment during the present study.
3. Patients with conditions that may preclude safe use of pitavastatin or ezetimibe, including: active liver disease or persistent elevations of serum transaminases (AST or ALT >3× ULN); severe renal impairment (eGFR <30 mL/min/1.73 m²) or end-stage renal disease on dialysis; uncontrolled hypothyroidism (TSH >10 mIU/L); active myopathy or unexplained CK elevation >5× ULN at baseline; rhabdomyolysis history; pregnancy, lactation, or planned pregnancy during the study period; and severe systemic illness expected to limit participation or survival during the 48-week observation period. Conditions covered by the LivaloZet^®^ product label contraindications are excluded under criterion 1) above.
4. Individuals who are considered to have difficulty participating in the study for other reasons based on the judgment of the investigator.

### 2.3 Cardiovascular risk classification

Cardiovascular risk classification (**Table 2**) is assigned by the treating investigator at Visit 1 (baseline), based on the patient’s clinical history, comorbidities, and available risk-factor data, in accordance with the 2022 KSoLA guidelines (9). If risk-factor data are incomplete for any participant, the investigator will assign the most conservative (higher-risk) category supported by available evidence, and the participant will be flagged for sensitivity analysis. Risk classification will not be re-assigned during follow-up; the baseline category determines target LDL-C used for goal-attainment analyses.

### 2.4 Efficacy endpoints

#### 2.4.1 Primary endpoint

Percent change from baseline in LDL-C level at Week 24 (calculated, see Section 2.6.4 for LDL-C measurement method).

#### 2.4.2 Secondary endpoints

1. Percent change from baseline in LDL-C level at Week 48.
2. Absolute change from baseline in LDL-C level at Week 24 and Week 48.
3. Percent change and absolute change from baseline in lipid parameters at Week 24 and Week 48: Total cholesterol (TC), triglyceride (TG), HDL-C, non-HDL-C. Apolipoprotein B (Apo B) is not a required measurement; it will be collected and analyzed descriptively only when available from routine clinical practice.
4. Achievement rate (%) for target LDL-C level at Week 24 and Week 48: Very high risk group <55 mg/dL, high risk group <70 mg/dL, diabetes group <100 mg/dL, moderate risk group <130 mg/dL, and low risk group <160 mg/dL according to the classification of cardiovascular disease risk groups.
5. Medication adherence (proportion of days covered, PDC), persistence (time from initiation to discontinuation), dose modifications, and treatment switching during the 48-week observation period.

#### 2.4.3 Safety endpoints

1. Adverse events (AEs), classified by severity (mild, moderate, severe), causality to study medication (related, possibly related, unrelated), and seriousness (serious vs. non-serious) according to ICH-E2A criteria.
2. Laboratory tests (blood chemistry), including systematic monitoring of AST, ALT, and creatine kinase (CK) at every visit for assessment of statin-related hepatotoxicity and myopathy. Pre-specified thresholds for safety signals are: AST or ALT >3× upper limit of normal (ULN); CK >5× ULN with muscle symptoms, or >10× ULN regardless of symptoms.
3. Vital signs.
4. Pre-specified adverse events of special interest: hepatotoxicity, myopathy, rhabdomyolysis, new-onset diabetes mellitus, and discontinuation due to AEs.

#### 2.4.4 Exploratory endpoints

1. Absolute change from baseline in blood glucose and glucose metabolism parameters at Week 24 and Week 48: Hemoglobin A1c (HbA1c), homeostasis model assessment of insulin resistance (HOMA-IR), homeostasis model assessment of β-cell function (HOMA-β).
2. Percent change and absolute change from baseline in high sensitivity C-reactive protein (hs-CRP) at Week 24 and Week 48.

Exploratory laboratory parameters (HbA1c, HOMA-IR, HOMA-β, hs-CRP) are collected when available from routine clinical practice and are therefore subject to non-random missingness. To mitigate this limitation, baseline characteristics will be compared between participants with and without available measurements, and inverse-probability-of-availability weighting will be considered as a sensitivity analysis when interpreting these endpoints.

### 2.5 Sample size

This study is an observational study designed to describe the LDL-C-lowering effectiveness and safety of LivaloZet^®^ Tablet in patients with dyslipidemia accompanied by metabolic syndrome. Rather than conducting formal hypothesis-based efficacy testing, the study aims to describe utilization, effectiveness, and safety patterns in a real-world clinical setting. The planned sample size of 10,000 participants was selected on the basis of the following considerations: (1) precision—a sample size of 10,000 with an anticipated dropout rate of approximately 20% yields an effective analysis population of ≥8,000 participants, providing a two-sided 95% confidence interval of approximately ±0.5% for the primary endpoint (percent change in LDL-C), assuming a standard deviation of 22%; (2) adverse event detection—this sample size provides ≥80% power to detect adverse events occurring at a true rate of ≥0.04% (approximately 1 in 2,500); (3) subgroup analyses—the planned sample size enables adequately powered descriptive analyses across pre-specified subgroups defined by cardiovascular risk category, prior lipid-lowering treatment history, dose (2/10 mg vs 4/10 mg), age, sex, and presence of diabetes; and (4) representativeness—a sample of this magnitude enrolled across multiple centers throughout Korea is expected to reasonably reflect the heterogeneity of the target population in routine clinical practice.

### 2.6 Statistical analysis

#### 2.6.1 Analysis populations

The full analysis set (FAS) includes all enrolled participants who received at least one dose of LivaloZet^®^ and have at least one post-baseline assessment. The per-protocol set (PPS) excludes participants with major protocol deviations. The safety analysis set (SAS) includes all participants who received at least one dose of LivaloZet^®^. The primary efficacy analysis will be performed on the FAS using last-observation-carried-forward (LOCF); the PPS will be analyzed as a sensitivity analysis.

#### 2.6.2 Statistical methods

All hypothesis tests are performed at a significance level of 5%, and two-sided tests are used unless otherwise specified. SAS (Version 9.4 or higher, SAS Institute, Cary, NC, USA) is used as the software for statistical analysis. The significance level is not adjusted for per-visit analyses. Continuous variables are summarized using mean ± standard deviation (or median with interquartile range for skewed distributions). Categorical variables are summarized using counts and percentages. Two-sided 95% confidence intervals are reported alongside all point estimates for the primary and key secondary endpoints. Center-level variation will be evaluated using random-effects models with center as a random intercept, where appropriate. For variables with potentially non-normal distributions (notably triglycerides and high-sensitivity C-reactive protein), normality will be assessed graphically (histograms, Q–Q plots) and using the Shapiro–Wilk test prior to analysis; in cases of substantial skewness, results will be summarized as median (IQR) and analyzed using nonparametric methods (Wilcoxon signed-rank test for within-participant change, Mann–Whitney U test for between-group comparisons), with sensitivity analyses on log-transformed values reported as supportive evidence.

#### 2.6.3 Subgroup analyses

Pre-specified subgroup analyses will be performed for the primary endpoint by: (1) cardiovascular risk category; (2) prior lipid-lowering treatment history (treatment-naïve vs inadequate response); (3) dose (2/10 mg vs 4/10 mg); (4) age (<65 vs ≥65 years); (5) sex; (6) presence of diabetes mellitus; and (7) baseline LDL-C (<130 vs ≥130 mg/dL). Subgroup analyses are descriptive; no formal interaction testing is planned.

#### 2.6.4 Handling of missing data

In the event of missing data, missing values for efficacy endpoints are imputed using the last observation carried forward (LOCF) method. However, baseline values are not carried forward to replace post-baseline values. Safety analyses are conducted using observed cases (OC) without imputation of missing data. LOCF was selected as the primary approach for efficacy endpoints because it is a widely accepted, conservative, and easily interpretable method in regulatory and observational research, and because in a single-arm descriptive study without between-group comparison the bias introduced by LOCF is generally toward attenuation of within-participant change rather than spurious between-group differences. We acknowledge, however, that LOCF assumes that the last observed value is representative of the subsequent (missing) value, which may not hold for participants who discontinue treatment due to lack of efficacy or adverse events; the multiple sensitivity analyses described below (observed cases, multiple imputation, MMRM) are therefore essential to evaluate the robustness of the primary findings in this 48-week observational setting. To address the potential bias introduced by LOCF, two sensitivity analyses will be performed: (i) observed-cases analysis without imputation; and (ii) multiple imputation under the missing-at-random assumption (m=20 imputations, predictive mean matching). Mixed-effects models for repeated measures (MMRM) including all available longitudinal observations will be conducted as an additional sensitivity analysis, with no imputation required. The robustness of the primary conclusion will be assessed by comparing point estimates and confidence intervals across these analytical approaches.

#### 2.6.5 Handling of confounding and selection bias

As treatment selection (dose choice) is made by the treating physician, confounding by indication is a potential concern when comparing outcomes across dose subgroups. Sensitivity analyses using propensity-score adjustment for baseline characteristics (age, sex, baseline LDL-C, cardiovascular risk category, prior lipid-lowering therapy, comorbidities) will be performed for dose-stratified analyses. Selection bias—arising from the voluntary nature of participation and the open-label design—is acknowledged as an inherent limitation and will be addressed by detailed reporting of consecutive screening logs, reasons for non-participation, and comparison of enrolled vs. screened-but-not-enrolled patients where data are available.

#### 2.6.6 Anticipated attrition and loss to follow-up

Based on prior Korean real-world observational studies of lipid-lowering therapy, the anticipated attrition rate at Week 24 is approximately 10–15%, and at Week 48 approximately 20–25%. Participants lost to follow-up will be retained in the FAS using LOCF for the primary analysis, with sensitivity analyses as described in Section 2.6.4. Reasons for discontinuation will be systematically captured (e.g., adverse event, lack of efficacy, patient withdrawal, switch to alternative therapy, loss to follow-up).

### 2.7 Laboratory measurements and quality control

Lipid panel (LDL-C, TC, TG, HDL-C, non-HDL-C) and safety laboratory tests (AST, ALT, CK) are performed at each participating center’s local laboratory using standard clinical assays. LDL-C may be measured either directly (by homogeneous direct assay) or calculated using the Friedewald formula. When TG ≥400 mg/dL, the Friedewald formula is invalid and direct LDL-C measurement is required; in such cases, non-HDL-C will be used as a supplementary measure. The LDL-C measurement method (direct vs calculated) will be recorded for every measurement and analyzed as a covariate. To address inter-laboratory variability, all participating laboratories must be certified under the Korean Laboratory Accreditation Scheme or equivalent national/international standards, and external quality assurance (EQA) results will be reviewed annually. Site-level training will be provided prior to study initiation, including standardized fasting requirements (≥12 hours preferred, ≥9 hours minimum for lipid and glucose testing), standardized variable definitions, and standardized data entry procedures. Electronic case report forms include built-in range and consistency checks. Independent monitoring visits will be conducted at high-enrolling centers, with remote source data verification at all sites. Discrepancies will be addressed through formal data queries.

### 2.8 Therapeutic lifestyle counseling

All participants receive standardized Therapeutic Lifestyle Change (TLC) counseling at Visit 1, Visit 2, and Visit 3, in accordance with the 2022 KSoLA guidelines. Counseling content includes recommendations on saturated-fat and cholesterol intake, increased physical activity (≥150 minutes/week of moderate-intensity activity), weight management, and smoking cessation. To minimize inter-center variability, all investigators receive standardized TLC training materials prior to study initiation. Adherence to TLC recommendations is assessed at each visit using a brief structured questionnaire and is recorded but is not adjudicated as a study endpoint.

## 3 Discussion

This study is a multi-center, prospective observational study designed to describe the LDL-C-lowering effectiveness and safety patterns associated with the use of LivaloZet^®^ Tablets (pitavastatin/ezetimibe fixed-dose combination) in patients with dyslipidemia accompanied by metabolic syndrome in real-world clinical settings in Korea. To our knowledge, real-world evidence on pitavastatin/ezetimibe fixed-dose combination therapy in Korean patients with dyslipidemia accompanied by metabolic syndrome remains limited. Specifically, no prospective observational study with a planned enrollment of this scale has been reported for the pitavastatin/ezetimibe fixed-dose combination specifically in Korean patients with dyslipidemia accompanied by metabolic syndrome; this characterization defines the scope of the novelty claim in the present protocol.Although several randomized controlled trials have evaluated the efficacy of pitavastatin/ezetimibe combination therapy in patients with hypercholesterolemia (24–26), real-world evidence in patients with concurrent dyslipidemia and metabolic syndrome remains scarce.

The clinical significance of managing dyslipidemia in patients with metabolic syndrome is well established. Metabolic syndrome, defined by the coexistence of abdominal obesity, hypertension, hyperglycemia, and atherogenic dyslipidemia, substantially amplifies the risk of cardiovascular events beyond that attributable to individual risk factors alone (5, 6). Epidemiological data from Korea indicate that the prevalence of dyslipidemia among adults aged ≥20 years was 37.5%, while that of metabolic syndrome among adults aged ≥19 years was 24.9%, both rising with age (7, 8). Given this epidemiological backdrop and the unmet need for achieving LDL-C targets in Korean patients with cardiovascular risk (27), effective and well-tolerated pharmacological strategies for lipid management in this high-risk population are urgently needed (9).

Statins remain the cornerstone of pharmacological lipid management, and their combination with ezetimibe has been well established as an effective and safe approach for patients who require potent LDL-C reduction or who cannot tolerate high-dose statin monotherapy (9, 11, 12). Prior randomized controlled trials provide the biological and clinical rationale for the present study. The landmark IMPROVE-IT trial demonstrated that adding ezetimibe to statin therapy significantly reduced major cardiovascular events compared with statin monotherapy, providing compelling evidence that non-statin LDL-C lowering translates into clinical benefit (24). Furthermore, the RACING trial confirmed that moderate-intensity statin combined with ezetimibe achieves non-inferior LDL-C reduction with fewer adverse events compared with high-intensity statin monotherapy (25). However, because the present study is a single-arm observational design without a comparator, it cannot replicate the comparative inferences of these randomized trials. Rather, the present study complements such trials by providing descriptive real-world data on routine clinical use. Among statins, pitavastatin is distinguished by its relatively neutral effect on glucose metabolism, compared with several other statins (10, 21–23). Meta-analyses and randomized trials have demonstrated that pitavastatin has a comparatively favorable profile with respect to fasting glucose, HbA1c, and insulin sensitivity, making it a particularly attractive option for patients with metabolic syndrome who frequently have impaired glucose metabolism or established type 2 diabetes (21–23). A recent phase III randomized trial in Korean patients confirmed that pitavastatin/ezetimibe combination therapy achieved superior LDL-C reduction of approximately 52-53% from baseline compared with pitavastatin monotherapy (37-45%), with a comparable safety profile (26). The fixed-dose combination of pitavastatin and ezetimibe in LivaloZet^®^ Tablets is thus a rationally designed therapeutic option that addresses both the lipid-lowering needs and the metabolic concerns of this patient population.

The primary endpoint of this study, the percent change in LDL-C from baseline to Week 24, was selected as the most clinically relevant measure of lipid-lowering effectiveness in routine practice. LDL-C reduction is a well-validated surrogate endpoint for cardiovascular risk reduction, and a substantial body of evidence confirms that each 1 mmol/L reduction in LDL-C corresponds to approximately a 22% reduction in major vascular events (1, 3, 4). The secondary endpoints, including LDL-C changes at Week 48, changes in other lipid parameters (TC, TG, HDL-C, and non-HDL-C; Apo B when available), and the rate of achieving guideline-recommended LDL-C targets, will provide a comprehensive lipid profile assessment over a 48-week treatment period. The achievement of target LDL-C levels stratified by cardiovascular disease risk group is of particular clinical importance, given that real-world data from Korea have consistently demonstrated suboptimal LDL-C goal attainment rates, especially in very high-risk patients (27). Importantly, however, this study does not assess major adverse cardiovascular events (MACE), myocardial infarction, stroke, revascularization, cardiovascular death, or hospitalization. Cardiovascular clinical outcomes are beyond the scope of this 48-week protocol and would require a substantially longer follow-up period and an active comparator to be interpretable; such outcomes should be the subject of future, appropriately designed studies.

Beyond lipid parameters, the exploratory endpoints pertaining to glucose metabolism (HbA1c, HOMA-IR, HOMA-β) and systemic inflammation (hs-CRP) are of considerable scientific interest. The inclusion of these exploratory endpoints reflects the unique metabolic profile of the target population and the well-characterized metabolic properties of pitavastatin (21–23). Pitavastatin has been reported in prior studies to have a comparatively neutral effect on glucose metabolism relative to several other statins (21–23). By descriptively capturing changes in insulin resistance and beta-cell function through HOMA-IR and HOMA-β, this study will describe real-world patterns of the metabolic safety of LivaloZet^®^ Tablets in patients with metabolic syndrome - data that are particularly relevant given that a substantial proportion of enrolled patients are expected to have concurrent pre-diabetes or diabetes. Because exploratory laboratory tests are collected only when available in routine practice, results should be interpreted with caution due to potential non-random missingness. Similarly, monitoring hs-CRP will allow an assessment of patterns in inflammatory markers during pitavastatin/ezetimibe combination therapy.

The observational design of the PIVOT study has inherent strengths and limitations that merit discussion. The principal strength of this design is its ability to capture the real-world use patterns and safety of LivaloZet^®^ Tablets under routine clinical practice conditions, reflecting a diverse and unselected patient population that is more representative of everyday clinical encounters than participants typically enrolled in randomized controlled trials. Observational studies with large sample sizes are increasingly recognized as a critical complement to randomized trials, especially for generating evidence in patient subgroups that are underrepresented in controlled settings, such as patients with multiple comorbidities or complex metabolic profiles. The large planned sample size of 10,000 participants enrolled across multiple centers will provide substantial statistical power for descriptive analyses and subgroup evaluations, and will improve the ability to detect uncommon adverse events. The multi-center design further enhances the generalizability of the findings across different clinical settings throughout Korea.

Nonetheless, the observational nature of this study introduces several important limitations that must be acknowledged. First and most importantly, the absence of a control or comparator arm precludes causal inference and means that the present study cannot establish superiority, non-inferiority, or comparative effectiveness of LivaloZet^®^ versus pitavastatin monotherapy, high-intensity statin monotherapy, ezetimibe combined with other statins, or lifestyle intervention alone, and the results must be interpreted in the context of potential confounding by indication, as treatment decisions are made at the discretion of the treating physician rather than by randomization. Patients prescribed different doses (2/10 mg vs 4/10 mg) or entering with different prior treatment histories may therefore differ systematically in baseline characteristics; pre-specified propensity-score adjusted sensitivity analyses (Section 2.6.5) will address this concern, but residual confounding by unmeasured variables cannot be excluded. Selection bias is a further concern: the voluntary nature of participation and the open-label design mean that participants who consent to enrollment and complete follow-up may differ systematically from the broader real-world target population, being potentially more adherent, more health-engaged, or healthier. Additionally, as optional laboratory assessments - including glucose metabolism parameters, hs-CRP, and ApoB - are collected only when available from routine clinical practice, the completeness of these data may vary across centers and participants, potentially introducing non-random missingness; sensitivity analyses (Section 2.6.4) will address this concern. AST, ALT, and CK, in contrast, are systematically collected at every visit given their clinical importance for statin safety. Although LOCF is a pragmatic approach widely employed in observational studies, it may not fully account for informative missingness; sensitivity analyses using observed cases, multiple imputation, and MMRM (Section 2.6.4) will be used to assess the robustness of the primary findings. Inter-laboratory variability—since lipid panels and safety laboratories are measured at participating centers’ local laboratories—is mitigated by laboratory accreditation requirements and EQA review (Section 2.7), but cannot be entirely eliminated. Finally, this study does not include a comparator arm using other statin/ezetimibe combinations, which limits direct comparative effectiveness inferences, nor does it include cardiovascular clinical outcomes (MACE, myocardial infarction, stroke, revascularization, cardiovascular death, hospitalization), which would require longer follow-up and a randomized comparison to be interpretable. These limitations are inherent to observational study designs and should be considered when interpreting the findings.

In conclusion, the PIVOT study will generate descriptive, prospective real-world evidence on the LDL-C-lowering effectiveness, lipid-target attainment, and safety patterns associated with the use of LivaloZet^®^ Tablets in a clinically relevant and growing patient population — those with both dyslipidemia and metabolic syndrome. The results of this study are expected to inform clinical decision-making in routine practice and to provide descriptive evidence on metabolic safety patterns in patients with concomitant glucose metabolism disorders (21–23). Importantly, the design of this study does not allow for the demonstration of comparative efficacy, non-inferiority, or cardiovascular benefit; such inferences will require future randomized controlled trials with appropriate comparators and clinical endpoints.

## References

[1] National Cholesterol Education Program (NCEP) Expert Panel . Third report of the NCEP Expert Panel on detection, evaluation, and treatment of high blood cholesterol in adults (Adult Treatment Panel III) final report. Circulation. (2002) 106:3143–421. doi: 10.1161/circ.106.25.3143 12485966

[2] Keys A Aravanis C Blackburn H Van Buchem FSP Buzina R Djordjevic BS . Probability of middle-aged men developing coronary heart disease in five years. Circulation. (1972) 45:815–28. doi: 10.1161/01.cir.45.4.815 5016014

[3] Linsel-Nitschke P Götz A Erdmann J Braenne I Braund P Hengstenberg C . Lifelong reduction of LDL-cholesterol related to a common variant in the LDL-receptor gene decreases the risk of coronary artery disease: a Mendelian randomisation study. PloS One. (2008) 3:e2986. doi: 10.1371/journal.pone.0002986 18714375 PMC2500189

[4] Ference BA Ginsberg HN Graham I Ray KK Packard CJ Bruckert E . Low-density lipoproteins cause atherosclerotic cardiovascular disease. 1. Evidence from genetic, epidemiologic, and clinical studies. A consensus statement from the European Atherosclerosis Society Consensus Panel. Eur Heart J. (2017) 38:2459–72. doi: 10.1093/eurheartj/ehx144 28444290 PMC5837225

[5] Lakka HM Laaksonen DE Lakka TA Niskanen LK Kumpusalo E Tuomilehto J . The metabolic syndrome and total and cardiovascular disease mortality in middle-aged men. JAMA. (2002) 288:2709–16. doi: 10.1001/jama.288.21.2709 12460094

[6] Bae JH Haam JH Jeon E Kang SY Song S Park CY . 2024 clinical practice guidelines for the diagnosis and pharmacologic treatment of overweight and obesity by the Korean Society for the Study of Obesity. J Obes Metab Syndr. (2025) 34:322–43. doi: 10.7570/jomes25042 40819841 PMC12583794

[7] Kwon O Lee SY Kim B Han K Ahn J . Dyslipidemia fact sheet in South Korea, 2024. J Lipid Atheroscler. (2025) 14:298–311. doi: 10.12997/jla.2025.14.3.298 41048599 PMC12488789

[8] Rhee EJ . Prevalence and current status of cardiometabolic risk factors in Korean adults based on Fact Sheets 2024. Endocrinol Metab (Seoul). (2025) 40:174–84. doi: 10.3803/enm.2025.2398 40312986 PMC12061748

[9] Yang YS Kim HL Kim SH Moon MK . Lipid management in Korean people with type 2 diabetes mellitus: Korean Diabetes Association and Korean Society of Lipid and Atherosclerosis consensus statement. Diabetes Metab J. (2023) 47:1–9. doi: 10.4093/dmj.2022.0448 36727160 PMC9925153

[10] Hoy SM . Pitavastatin: a review in hypercholesterolemia. Am J Cardiovasc Drugs. (2017) 17:157–68. doi: 10.6515/ACS20160611A 28130659

[11] Ramkumar S Raghunath A Raghunath S . Statin therapy: review of safety and potential side effects. Acta Cardiol Sin. (2016) 32:631–9. doi: 10.1016/j.hlc.2015.06.184 27899849 PMC5126440

[12] Grundy SM Stone NJ Bailey AL Beam C Birtcher KK Blumenthal RS . 2018 AHA/ACC/AACVPR/AAPA/ABC/ACPM/ADA/AGS/APhA/ASPC/NLA/PCNA guideline on the management of blood cholesterol: a report of the American College of Cardiology/American Heart Association Task Force on Clinical Practice Guidelines. Circulation. (2019) 139:e1082–e1143. doi: 10.1161/cir.0000000000000625 30586774 PMC7403606

[13] Jones PH Davidson MH Stein EA Bays HE McKenney JM Miller E . Comparison of the efficacy and safety of rosuvastatin versus atorvastatin, simvastatin, and pravastatin across doses (STELLAR trial). Am J Cardiol. (2003) 92:152–60. doi: 10.1016/s0002-9149(03)00530-7 12860216

[14] Dujovne CA Ettinger MP McNeer JF Lipka LJ LeBeaut AP Suresh R . Efficacy and safety of a potent new selective cholesterol absorption inhibitor, ezetimibe, in patients with primary hypercholesterolemia. Am J Cardiol. (2002) 90:1092–7. doi: 10.1016/s0002-9149(02)02798-4 12423709

[15] Davidson MH McGarry T Bettis R Melani L Lipka LJ LeBeaut AP . Ezetimibe coadministered with simvastatin in patients with primary hypercholesterolemia. J Am Coll Cardiol. (2002) 40:2125–34. doi: 10.1016/s0735-1097(02)02610-4 12505224

[16] Kerzner B Corbelli J Sharp S Lipka LJ Melani L LeBeaut AP . Efficacy and safety of ezetimibe coadministered with lovastatin in primary hypercholesterolemia. Am J Cardiol. (2003) 91:418–24. doi: 10.1016/s0002-9149(02)03236-8 12586255

[17] Melani L Mills R Hassman D Lipetz R Lipka LJ LeBeaut AP . Efficacy and safety of ezetimibe coadministered with pravastatin in patients with primary hypercholesterolemia: a prospective, randomized, double-blind trial. Eur Heart J. (2003) 24:717–28. doi: 10.1016/s0195-668x(02)00803-5 12713766

[18] Ballantyne CM Houri J Notarbartolo A Melani L Lipka LJ Suresh R . Effect of ezetimibe coadministered with atorvastatin in 628 patients with primary hypercholesterolemia: a prospective, randomized, double-blind trial. Circulation. (2003) 107:2409–15. doi: 10.1161/01.CIR.0000068312.21969.C8 12719279

[19] Goldberg AC Sapre A Liu J Capece R Mitchel YB . Efficacy and safety of ezetimibe coadministered with simvastatin in patients with primary hypercholesterolemia: a randomized, double-blind, placebo-controlled trial. Mayo Clin Proc. (2004) 79:620–9. doi: 10.4065/79.5.620 15132403

[20] Bays HE Ose L Fraser N Tribble DL Quinto K Reyes R . A multicenter, randomized, double-blind, placebo-controlled, factorial design study to evaluate the lipid-altering efficacy and safety profile of the ezetimibe/simvastatin tablet compared with ezetimibe and simvastatin monotherapy in patients with primary hypercholesterolemia. Clin Ther. (2004) 26:1758–73. doi: 10.1016/j.clinthera.2004.11.016 15639688

[21] Vallejo-Vaz AJ Kondapally Seshasai SR Kurogi K Michishita I Nozue T Sugiyama S . Effect of pitavastatin on glucose, HbA1c and incident diabetes: a meta-analysis of randomized controlled clinical trials in individuals without diabetes. Atherosclerosis. (2015) 241:409–18. doi: 10.1016/j.atherosclerosis.2015.06.001 26074315

[22] Yamakawa T Takano T Tanaka S Kadonosono K Terauchi Y . Influence of pitavastatin on glucose tolerance in patients with type 2 diabetes mellitus. J Atheroscler Thromb. (2008) 15:269–75. doi: 10.5551/jat.e562 18981652

[23] Gumprecht J Gosho M Budinski D Hounslow N . Comparative long-term efficacy and tolerability of pitavastatin 4 mg and atorvastatin 20–40 mg in patients with type 2 diabetes mellitus and combined (mixed) dyslipidaemia. Diabetes Obes Metab. (2011) 13:1047–55. doi: 10.1111/j.1463-1326.2011.01477.x 21812889

[24] Cannon CP Blazing MA Giugliano RP McCagg A White JA Theroux P . Ezetimibe added to statin therapy after acute coronary syndromes. N Engl J Med. (2015) 372:2387–97. doi: 10.1056/nejmoa1410489 26039521

[25] Kim BK Hong SJ Lee YJ Hong SJ Yun KH Hong BK . Long-term efficacy and safety of moderate-intensity statin with ezetimibe combination therapy versus high-intensity statin monotherapy in patients with atherosclerotic cardiovascular disease (RACING): a randomised, open-label, non-inferiority trial. Lancet. (2022) 400:380–90. doi: 10.1016/s0140-6736(22)00916-3 35863366

[26] Oh SJ Kim BJ Her SH Han KH Cha DH Jo SH . Multicenter, randomized, double-blind, active-controlled, factorial design, phase III clinical trial to evaluate the efficacy and safety of combination therapy of pitavastatin and ezetimibe versus monotherapy of pitavastatin in patients with primary hypercholesterolemia. Clin Ther. (2022) 44:1310–25. doi: 10.1016/j.clinthera.2022.09.001 36241463

[27] Yang YS Yang BR Kim MS Hwang Y Choi SH . Low-density lipoprotein cholesterol goal attainment rates in high-risk patients with cardiovascular diseases and diabetes mellitus in Korea: a retrospective cohort study. Lipids Health Dis. (2020) 19:5. doi: 10.1186/s12944-019-1158-5 31926562 PMC6954559

